# Precision Medicine: Transforming Cancer Research through Targeted Therapies

**DOI:** 10.2174/0113892029357275250721125929

**Published:** 2025-07-29

**Authors:** Satyam Kumar Agrawal, Sushmita Sunil Jain, Madhunika Agrawal

**Affiliations:** 1 Centre for in vitro Studies and Translational Research, Chitkara School of Health Sciences, Chitkara University, Rajpura, Punjab, 140401, India;; 2 Cellsinvitro Lifesciences Pvt. Ltd., SAS Nagar, Punjab, 140308, India

**Keywords:** Precision medicine, cancer research, immunotherapy, biomarkers, CTCs

## Abstract

Precision medicine is a landmark strategy that has been changing the future of health care through matching treatment plans with each individual patient’s needs and requirements. It permits the discovery of certain genetic abnormalities that cause tumors in cancer research, resulting in tailored medicines and better outcomes. The new drug development process is facilitated by precision medicine, focusing on biomarkers and patient classification because they allow for faster identification of new treatments. Emerging trends in omics technologies and Artificial Intelligence for data processing have patient-centered telemedicine applications. Ethical and privacy issues are addressed, focusing on data security and informed consent. The additional development of precision medicine offers hope for bridging gaps in healthcare delivery systems, addressing rare disease challenges, and promoting global healthcare initiatives. The revolutionizing nature of healthcare and improved patient outcomes can only be fully realized through acceptance and support of precision medicine to its fullest extent. This review evaluates various applications of precision medicine with an emphasis on how it could potentially change the paradigm of cancer research.

## 1 INTRODUCTION

Precision medicine is essentially built on a molecular understanding of diseases and acknowledges that the genetic composition of a patient affects their overall prognosis, susceptibility to different diseases, and their response towards treatment. Being a patient-centric approach, it involves patients at almost every step from diagnosis to treatment outcomes, along with interview sessions for long hours with follow-up discussions. Thus, healthcare is no longer provided in a one-size-fits-all manner but rather aims at tailoring therapeutic approaches based on the specific genomic alterations present in each patient’s disease etiology. By identifying mutations, gene expressions and molecular signatures, it becomes realistic to select treatments which target the causative agents of cancer, leading to more powerful and potentially safer interventions. Precision medicine helps us understand various cancer subtypes through analyzing the genome and transcriptome of tumors, facilitating the identification of vulnerabilities that can be exploited as targets for therapy [1]. The discovery of reliable biomarkers for genetic aberrations, protein expression or even tumor micro-environmental aspects, enables more focused and efficient interventions by enhancing diagnosis and providing suggestions for treatment choices [2]. Furthermore, the advances in technology, such as high-throughput techniques including next-generation sequencing (NGS) and bioinformatics, have helped researchers and physicians to delve deeper into the genomic landscape, thereby leading to a better understanding of diseases, most importantly cancer [3].

Bioinformatics tools can be helpful in integrating, analyzing and interpreting OMICS data as well as clinical images to have a better perspective of the mechanisms involved in the pathological state. An accurate nuclear segmentation is a crucial task in histopathological study owing to the variable nature of nuclei and cellular morphology. An AI-assisted study was conducted on histological specimens. With the help of The Cancer Genome Atlas and Triple-Negative Breast Cancer datasets, the accurate nuclear segmentation in images was investigated and significant improvement was observed in segmentation [4]. In a study, with the help of bioinformatics, the association between NIMA-related kinase (NEK) family genes and breast cancer progression was determined [5]. In another study, expression profiles of GSE9750, GES7803, GES63514, GES17025, GES115810, and GES36389 were extracted from Gene Expression Omnibus to employ them to investigate differential gene expression between cancer and normal tissues [6]. In a similar study, a dataset of eight microarrays related to lung cancer was meta-analysed to understand differentially expressed genes and interactions between proteins. A potent regulatory association between CDK1 and HSP90AA1 was observed in non-small cell lung cancer [7].

In contrast to conventional chemotherapy, which is nonspecifically cytotoxic towards rapidly dividing cancerous cells as well as to normal cells, targeted therapies aim at specifically interfering with signaling pathways that activate tumor growth [8, 9]. Researchers have used genomic analysis to generate important findings, which resulted in tailored treatments aimed at suppressing specific biomolecules implicated in cancer progression. The efficacy of these targeted therapies can be seen in the case of Imatinib, a tyrosine kinase inhibitor, which specifically inhibits BCR-ABL fusion protein, a known oncogenic driver in chronic myeloid leukemia. Imatinib has thus revolutionized treatment by being more precise and efficient than ordinary chemotherapy, as it minimizes side effects while increasing its efficacy [10]. This framework of combining genomic and genetic technologies in cancer care has made genetic testing an indispensable tool for oncologists.

There is a possibility that germline mutations can be detected by genetic testing. Genetic counseling can be helpful for people who have a family history of cancer, or specific risk factors that make them susceptible to cancer, which shall enable all such candidates to make informed choices regarding screening, prevention and treatment options [11]. Notably, genomic technologies in precision medicine are not without challenges. Financial implications of genomic analysis, as well as complex interpretation of the intricate genetic data and the associated ethical problems of genetic testing, have to be looked into. Precision medicine calls for responsible implementation that prioritizes equal access and addresses concerns on privacy and genetic discrimination [12]. Hence, this review focuses on key principles behind precision medicine by emphasizing genetics and genomics in advancing cancer diagnosis and treatment, as well as challenges with future prospects thereafter.

This review was written by conducting a literature search using PubMed, Scopus, and Google Scholar databases for studies published between 2010 and 2025, with very few references included from earlier years (1998-2004), where relevant. We used the keywords in the search engines, including “precision medicine”, “targeted therapy”, “cancer genomics”, “biomarkers”, “liquid biopsy”, “immunotherapy”, “ctDNA”, “CAR-t”, and “personalized oncology”. Articles were included according to their relevance, scientific brilliance, and role in advancing the understanding of precision medicine in oncology. Peer-reviewed original research, clinical trials, meta-analyses, and high-impact reviews were prioritized that highlight the applications of genomic and bioinformatic tools in cancer diagnostics, treatment, and management.

## 2 ROLE OF GENETICS IN CANCER: UNRAVELLING MECHANISMS OF ABNORMAL CELL PROLIFERATION

Frequently, cancer showcases a genetic basis, seeing that it is an intricate complex of diseases where there is abnormal multiplication of cells beyond normal control. Thus, any changes in the exact regulation of cell division and apoptosis could be attributed to mutations occurring in genes, thereby facilitating the tumor onset and advancement [13-16]. It is crucial to understand fully the genetic alterations underlying cancer development and progression so as to come up with precise and effective therapeutic strategies [17].

Genetic mutations are categorized into two main groups: germline and somatic mutations. Germline mutations are genetically inherited alterations that exist ubiquitously in all cells of an individual’s body, thereby conferring a predisposition to develop cancer. On the other hand, somatic ones result either spontaneously or due to some environmental factors, hence promoting neoplasm advancement. The interplay between germline and somatic mutations significantly contributes to the different phenotypic characteristics and treatment responses seen in patients with similar types of cancer [18].

Genetics primarily concerns itself with the study of individual genes and their respective functions. On the other hand, genomics adopts a more comprehensive approach by investigating the complete collection of genes, commonly referred to as the genome [19]. This means that genomics encompasses all genetic information contained in an organism’s DNA, including both coding and non-coding regions. Genomic studies provide a global view of genetic alterations driving cancer, thus shedding light on intricate networks and pathways controlling aberrant cell growth. Technological advancements have greatly boosted genomic research through the facilitation of whole genomes or important parts sequencing at an affordable price and within a short time [20]. For instance, the use of whole-exome sequencing and whole-genome sequencing makes it easy to find out about mutations, copy number variations, as well as structural rearrangements in order to fully discover the complexity of cancer genetics [21].

Whole genome sequencing (WGS) alone or together with whole transcriptome sequencing (WTS) has emerged as a valuable and unbiased representation of both pediatric as well as adult cancer profiling [22]. The importance of whole-genome and transcriptome sequencing (WTGS) consists of a detailed landscape of all alterations, including germline variants or structural variants, point mutations, fusion genes and tumor microenvironment. When compared with present genomic testing tools available, which mainly concentrate on a few numbers of genes tested alone or with targeted panels, WTGS offers unique opportunities to diagnose, verify and report a promisingly larger view of alterations, which can be attended with much sought-after and customized therapeutic impact [23]. A recent report demonstrating the genomic analysis was conducted on 189 tumor samples of different tumor types. It showed long-read genomic analysis exhibited potent advancements in understanding the structural variants, viral integration, DNA methylation, and information related to alleles necessary for cancer progression and prognosis [24]. In another study, whole-genome analysis conducted on varied diseases, including cancer, 20 patients with preliminary neoplasia, prostate and renal cancer, pancreatic cancer, *etc*., were diagnosed. Noninvasive whole-body tests as well as clinical testing covering the functioning of the liver, kidney, blood samples, endocrine, immune system, and lipid were employed. Moreover, it was important to notice that many patients were prone to cancer that were not even having any past family history or phenotypes at the time of testing [25]. Another analysis was performed with blood samples from adults of both genders, not having any illness or unexplained symptoms, and cancer, where four participants were recognized as having a risk of early-stage neoplasia that required urgent action [26].

The potential role of WGA in tailored therapy for cancer patients has been displayed in the study conducted on samples from 570 patients having advanced or metastatic cancer of different types. Personalized OncoGenomics program was employed where DNA- and RNA-based data isolated from biopsy samples was combined to produce full WGTA profiles. Medically applicable targets were recognized for more than 80% patients, out of which 37% were administered with WGTA-instructed treatments through variable modes, including clinical trials, off-label use and as standard therapies. RNA expressions and genome data amalgamation lead to more than 45% of treated patients with witnessed positive clinical benefit [27]. WTS of 294 formalin-fixed paraffin-embedded tissue samples isolated from the lung of varied cancers, namely primary cancer types, metastasis types, sarcoidosis, tuberculosis, and healthy tissues, were studied in a different study. Wilcoxon test followed by *p*-value correction *via* Benjamini-Hochberg was utilized to recognize Genes that have significantly different expression levels. This helps in precise histopathological tissue classification. Furthermore, mRNA expression of altered Tyrosine Kinase Inhibitors and antibody-drug conjugates was observed and claimed to be beneficial in guiding targeted therapies [28].

## 3 BIOMARKERS: INDICATORS IN THE GENOMIC TERRAIN

Biomarkers are crucial in precision medicine, which are measurable indications of several biological processes to detect diseases or conditions. Within cancer, genomic biomarkers are essential because they guide physicians by giving them useful information on which they can base individualized treatment plans. Not only does this application have an effect on increasing the accuracy of diagnosis for cancers, but it also enables understanding whether a particular therapeutic approach could be effective [29].

One well-known example includes the application of HER2 statuses with respect to breast cancer [30]. These tumors have high levels of expression for the HER2 protein, and thus, it is significantly responsive to therapy such as that of Trastuzumab [31, 32]. Treatment plans can therefore be guided by this marker towards meeting the specific needs of patients suffering from HER2-positive breast cancer; hence making such therapies more effective [33].

**Fig. (1):**
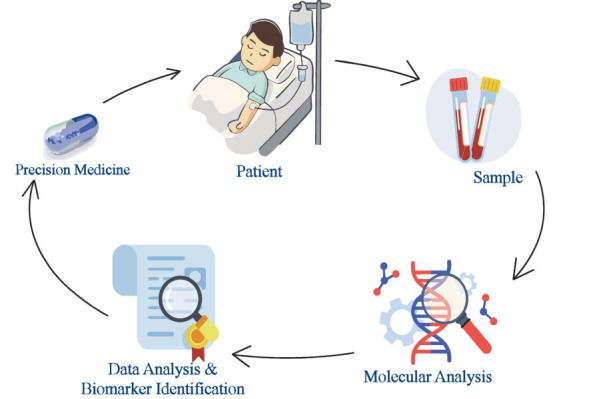
Biomarker identification for precision medicine.

Moreover, liquid biopsies, popularly referred to as blood-based biopsies, using circulating tumor DNA (ctDNA) and circulating tumor cells (CTCs) as circulating biomarkers, are a flexible way to understand the genomic complexities and possible directions underlying precision medicine [34]. Liquid biopsies are the analysis of various components within body fluids to ascertain about the health condition of a patient, particularly in relation to cancer. Liquid biopsy is a repeatable approach that helps monitor genomic characteristics of tumors in real time unlike conventional tissue biopsy methods which require several invasive procedures (Fig. **1**) [35].

### 3.1 ctDNA: Tracing Genetic Markers

Relatively, liquid biopsies comprise ctDNA as a vital component. The discharge of DNA fragments into the bloodstream is one of the natural life processes for tumors. ctDNA consists of some genetic content which is particular to genomic alterations in a tumor. It provides important information about the genetic makeup of the tumor by identifying mutations, copy number variations and other genomic aberrations that occur within the ctDNA. There are several ways in which this can be done with the use of ctDNA [36]. Due to its high sensitivity and specificity, it can help diagnose malignancies from an early stage and follow up on their progress throughout the disease. This is useful, especially when traditional biopsy methods are not available [37]. In this context, however, much attention and research have focused on ctDNA due to its potential clinical usefulness in precision medicine. Since ctDNA has been shown to identify minor residual diseases or early-stage malignancies, this offers great hope for rapid intervention and has been proven to be very useful in early diagnosis of lung, colorectal or breast cancers among others through several trials [38].

### 3.2 CTCs: Prognostic Utilization

The liquid biopsy concept goes beyond the ctDNA to encompass CTCs examination. CTCs are cancerous cells that have detached from primary tumor then migrated into the bloodstream. These rare cells carry important information on the biological characteristics of the tumor, its ability to invade tissues and the chance of metastasis taking place. Cancer progression is crucial for metastasis and it carries great significance for prognosis and overall health of patients [39].

Cancer advancement involves several stages; however, metastasis determines its severity positively, hence predicting its outcome. The presence of CTCs as precursors for metastatic lesions presents an opportunity for prognostication, incremental surveillance, *etc*. Advancements in knowledge regarding the molecular nature of CTCs have facilitated improved targeted therapies, thus reducing or managing metastatic disease [40]. CTCs provide critical information on how aggressive the tumor is. The elevated numbers are correlated with worse prognosis across different types of cancers and hence CTC numbers are useful for risk stratification. This data assists physicians in developing individualized treatment plans based on projected disease progression [41].

The examination of changes in amounts and features of CTCs through the course of treatment provides important information on the effectiveness of therapeutic interventions. The employment of real-time monitoring allows for timely adjustments to treatment regimens, thus augmenting their efficacy [42]. As technologies advance and more is learnt about genetic intricacies, liquid biopsies can form crucial tools for personalizing cancer treatments, individually targeted towards patients [43]. However, although liquid biopsies hold great promise, there are still ongoing challenges that need to be overcome. There is a need to enhance sensitivity and specificity, standardize methods used, and interpret complex genomic data. Additionally, it is essential to take into consideration the cost-effectiveness as well as accessibility regarding different patient populations in regard to liquid biopsy technology [44].

## 4 APPLICATIONS OF PRECISION MEDICINE IN SPECIFIC CANCER TYPES

The treatment of cancer has changed dramatically with the onset of precision medicine. Rather than using generalized treatments, it adjusts for the distinct genetic composition in every patient’s cancer. This section discusses tangible observations using precision medicine in various cancers:

### 4.1 Breast Cancer with HER2 Positivity

The turning point for managing HER2-positive breast cancer came when trastuzumab was introduced. The overactive HER2 protein is targeted by this specific therapy leading to marked improvements among patients suffering from this aggressive subtype. By investigating the genomic profiling of patients who have had remarkable responses to trastuzumab, case studies highlight the importance of precision medicine in guiding treatment decisions [45]. Besides, endocrine therapy is an instance of how precision medicine works, as it plays a fundamental role in treating hormone receptor-positive breast cancer. Genomic assays such as Oncotype DX and MammaPrint facilitate patient stratification regarding recurrence risk, which makes it possible for clinicians to appropriately adjust the level of endocrine therapy so that low-risk patients are not unnecessarily treated and high-risk cases receive more intensive interventions [46].

### 4.2 Non-Small Cell Lung Cancer

The discovery of EGFR mutations in non-small cell lung cancer (NSCLC) opened the door for the development of targeted treatments like gefitinib and erlotinib. Case studies elucidate scenarios where patients with mutated EGFR tumors experienced impressive responses to these drugs, enduring lengthy periods of disease control. In recent explorations, researchers have sought to increase the robustness of responses by unraveling intricacies of resistance mechanisms [47]. Moreover, identification of anaplastic lymphoma kinase (ALK) rearrangements in NSCLC led to the development of targeted treatments such as crizotinib. Clinical vignettes narrate how a new era opened up after ALK inhibitors were introduced, with tales of uncommonly long durations in prior gloomy prognoses [48].

Lorlatinib, a third-generation inhibitor of ALK, demonstrates significant antitumor activity in advanced ALK-positive NSCLC, particularly after prior ALK inhibitor failure. It shows marked intracranial efficacy, beneficial for patients with brain metastases, and leads to longer progression-free survival compared to crizotinib. Additionally, it improves quality of life despite a higher incidence of certain adverse events. As a result, lorlatinib is becoming a standard treatment option, offering hope for better outcomes in a challenging patient population [49]. Furthermore, a phase III CROWN study found that lorlatinib significantly outperformed crizotinib in progression-free survival for advanced ALK-positive non-small cell lung cancer, with a median follow-up of 60.2 months, showing progression-free survival rates of 60% for lorlatinib *versus* 8% for crizotinib after five years [50].

### 4.3 Colorectal Cancer

The molecular complexity of colorectal cancer is met with precision medicine, especially in cases involving RAS mutations. Anti-EGFR treatments, like cetuximab and panitumumab, work well against tumors with wild-type RAS but are ineffective against those with mutant RAS [51]. A recent study evaluated trastuzumabderuxtecan in patients with pretreated HER2-positive metastatic colorectal cancer. The study demonstrated promising antitumor activity of trastuzumabderuxtecan, including those with RAS mutations or previous anti-HER2 therapy [52].

A subgroup of colorectal cancers that are amenable to immune checkpoint inhibitors, such as pembrolizumab, is identified by mismatch repair deficiency and microsatellite instability (MSI-H). Some patients with MSI-H tumors, which were thought to be refractory, responded profoundly and long-lastingly to immunotherapy, as demonstrated by the clinical vignettes. Current research endeavors investigate the wider suitability of immunotherapy concerning various molecular subtypes [53]. Alternatively, adoptive cell immunotherapy emerged as a probable substitute to the immunotherapeutic approach for metastatic colorectal cancer (mCRC) patients through inducing immune active cells. Furthermore, a recent open-label, randomized, controlled, phase 3 trial reported a safer and improved result by combining PD1-T (novel PD‐1 blockade‐activated DC-CIK cells) cells with capecitabine plus oxaliplatin and bevacizumab (anti-VEGF-A Ab) in terms of progression-free survival and overall survival when compared to drugs alone for mCRC [54].

### 4.4 Melanoma with BRAF Mutation

A new era of precision therapeutics was brought about by the identification of BRAF mutations in melanoma. BRAF inhibitors, namely vemurafenib and dabrafenib, exhibit notable efficacy in treating BRAF-mutant melanomas, as evidenced by their remarkable response rates. However, the issue of acquired resistance continues to pose significant challenges [55]. Current studies explore combination approaches with BRAF inhibitors of the next generation to overcome these obstacles and increase response durability. A recent trial evaluated the combined efficacy of encorafenib (a BRAF inhibitor) and binimetinib (a MEK inhibitor) in treating BRAF-mutant melanoma. The trial demonstrated the improved progression-free survival rates compared to other treatments, and emphasized the importance of targeted therapies in improving patient outcomes [56].

### 4.5 Immunotherapy and Precision Medicine

The field of cancer treatment has experienced a significant transformation with the advent of immunotherapy, an innovative strategy that utilizes the body's immune system to target and eliminate cancerous cells. The convergence of immunotherapy and precision medicine presents a paradigm shift in the medical field. The amalgamation of immunotherapy and precision medicine, specifically examining immune checkpoint inhibitors and chimeric antigen receptor T-cell (CAR-T) therapies, is summarised in Fig. (**2**) [57].

### 4.6 Immunotherapy: Harnessing the Potency of the Immune System

The immune system relies on immunological checkpoints to effectively regulate its response to antigens, hence maintaining a state of equilibrium. Nevertheless, some types of malignancies take advantage of these regulatory checkpoints to avoid detection by the immune system. Immune checkpoint inhibitors (ICIs) serve to impair the evasion mechanism employed by cancer cells, thereby revitalizing the immune response against them. The key proteins that play a crucial role in regulating immune responses are programmed cell death protein 1 (PD-1), programmed death-ligand 1 (PD-L1), and cytotoxic T-lymphocyte-associated protein 4 (CTLA-4) [58].

### 4.7 Immune Checkpoint Inhibitors (ICIs)

Immunotherapy-based cancer interventions have exhibited notable efficacy in several types of malignancies. According to Havel *et al.* (2019), pembrolizumab and nivolumab, the anti-PD-1 antibodies and ipilimumab which target CTLA-4, have received regulatory approval for use as treatments against different malignancies such as melanoma, lung cancer and renal cell carcinoma [59]. Choosing patients who might respond well to immune checkpoint inhibitors (ICIs) through precision medicine is an important factor in increasing their efficiency, based on the molecular characteristics of tumors. Accordingly, unraveling the molecular nature of cancer allows for the development of specific therapeutic approaches that effectively target the underlying genetic abnormalities responsible for the disease, thereby minimizing harm done to normal cells [60].

ICIs have markedly changed the whole paradigm of complete cure of advanced and metastatic cancers in the past decade. PD-L1 expression and tumor mutational burden (TMB) have appeared as crucial biomarkers for predicting ICI response. A retrospective cohort study displayed that TMB more than or equal to 16 is positively correlated to the best ICI treatment results, in terms of higher patient survival rates and delayed disease progression, in NSCLC patients undergoing ICI treatment [61]. Furthermore, higher PD-L1 tumor expression is related to better all-cause survival, mainly due to a reduction in systemic mortality. Additionally, patients with high PD-L1 levels exhibited long-term survival, stressing the potent significance of multimodal therapy in such patients [62].

### 4.8 Chimeric Antigen Receptor T-cell (CAR-T) Therapies

CAR-T cell therapy involves the genetic modification of a patient's T cells to express chimeric antigen receptors (CARs) capable of identifying antigens found in cancer cells. The use of a precision-engineered strategy can augment the immune system's efficacy in specifically identifying and eliminating malignant cells [63]. CAR-T treatments, such as Kymriah and Yescarta, have exhibited remarkable efficacy in the treatment of hematological malignancies, specifically leukemia and lymphoma. Nevertheless, there are ongoing issues that need to be addressed, such as effectively managing the occurrence of cytokine release syndrome and neurotoxicity. The field of precision medicine assumes a crucial role in the anticipation and control of these adverse effects through the identification of patients with elevated susceptibility [64].

### 4.9 Personalized Care for Patients with Cancer

Successful implementation of precision medicine in oncology requires the joint efforts of researchers, health care professionals, as well as palliative care takers. Cancer diagnosis and subsequent cancer treatment have a huge effect on the mental health of the patient, along with his or her physical well-being. Depression and anxiety may hinder cancer treatment and recovery, as well as quality of life and survival. Cholecystokinin is a gastric system associated peptide hormone, having receptors expressed in various parts of CNS. It excretes digestive enzymes and supports in pancreatic growth. It is involved in regulating appetite, anxiety, and dopamine-related behaviour. Hyperexpressed cholecystokinin receptors are observed in precancerous pancreatic epithelial cells [65]. Cholecystokinin receptors have also been documented in many gastric malignancies, including gallbladder cancer, pancreatic cancer or colon cancers, and their blockade helps in the reduction of cancer cell growth as well as metastasis [66-68]. Similarly, long non-coding RNA LSAMP-1 gene polymorphism is associated with severe depression and panic attacks [69]. It was observed to be significantly reduced in lung cancer tissues of 170 cancer patients, and associated with poor prognosis [70]. Hence, modulating these genes might be beneficial in helping patients suffering from depression or anxiety-related panic attacks, thus improving patient’s quality of life.

### 4.10 Current Research: Creating the Path Forward

Continuing research efforts are geared towards revealing new levels of complexity as precision medicine keeps pushing the limits of cancer treatment. The core of these efforts is liquid biopsies, bioinformatics, integrative omics techniques, and comprehensive genomic profiling. Understanding the interaction of host factors, the tumor microenvironment, and genomic alterations drives the search for more targeted and efficient interventions in breast, lung, colorectal, and melanoma cancers [71].

A paradigm shift towards individualized and successful interventions is best illustrated using precision medicine in the treatment of melanoma, lung, colorectal, and breast cancers. Case studies highlight the revolutionary effects of immunotherapies and targeted therapies, providing windows into a future in which cancer treatment options will be as varied as the genomic landscapes they cover. Continuous research projects, marked by creativity and teamwork, hold the potential to open new avenues and push the limits of precision medicine in the never-ending quest for better patient outcomes [72].

## 5 CHALLENGES AND LIMITATIONS

**Fig. (3):**
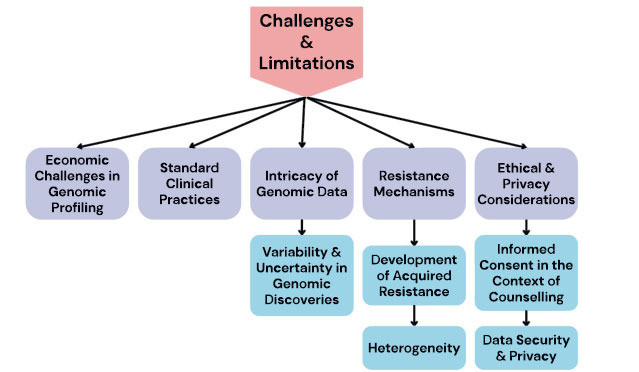
Challenges and limitations in precision medicine.

Precision medicine, which is widely regarded as a transformative approach in the field of healthcare, utilizes genomic profiling to customize medical interventions according to the specific needs of individual patients. However, the implementation of precision medicine is accompanied by a multitude of challenges and limitations, despite its transformative potential. The following discourse examines a range of challenges associated with the pursuit of precision medicine. It includes ethical and privacy concerns, as it explores various aspects, such as, access to genomic profiling and the deciphering of complex resistance mechansms (Fig. **3**) [73].

### 5.1 The Economic Challenges Associated with Genomic Profiling

There is still a large gap in access to genetic profiling, even with the quick advancement of genomic technologies. Financial barriers to certain sections of society are also a significant impediment, since the costs of sequencing technology act as a deterrent to mass adoption. The existence of an economic disparity raises questions about equitable and equal access to precision medicine's benefits, which may exacerbate the inequities in health that are already present [74].

### 5.2 Incorporation into Standard Clinical Practice

The problem of incorporating genetic profiling into everyday clinical practice is complex with many facets. Clinicians can find it difficult to interpret complicated genetic data, while incorporating these discoveries into decision-making processes necessitates drastic changes in medical education and healthcare delivery systems. The establishment of standardized guidelines will also be necessary so that there can be consistent use and efficacious application of genetic data in clinical operations [75].

### 5.3 The Intricacy of Genomic/somatic Data

The primary bottleneck that precision medicine needs to overcome is the intricate nature of genome data. It has to go through some accurate, precise and detailed bioinformatics tools and software. Detailed knowledge of this tool is needed in order to interpret the importance of genetic differences. The importance of understanding these variations can be useful when identifying relevant targets [76]. Additionally, reference sample deficiency, to generate optimized DNA datasets for marking the standardized performance of various algorithms, restricts the execution and analysis of cancer genomics. Furthermore, appropriate identification of mutations in somatic cells is crucial for genomic analysis and customized therapeutics because of variable allelic frequency, tumoral heterogeneity, frequent alterations in copy number and chromoanagenesis. Lack of authenticated and publicly available reference samples and reference datasets for somatic mutations also adds a barrier to error-free analysis [77].

### 5.4 The Presence of Variability and Uncertainty in Genomic Discoveries

The complexities associated with genetic findings create more complications during the interpretation of genomic data. This situation arises from variants of uncertain significance and a changing understanding of the functional significance of specific genetic alterations. In fact, navigating this state of uncertainty is important for conveying reliable information to both patients and clinicians [78].

## 6 RESISTANCE MECHANISMS: AN ADAPTATION TO EVOLUTIONARY DYNAMICS

### 6.1 The Development of Acquired Resistance

Resistance mechanisms need to be understood and overcome for cancer therapy. Tumors have the capacity to change with time, leading to new resistance, since treatments that worked before will not always work. The paradigms of precision oncology have evolved in clarifying the molecular basis of acquired resistance and designing mechanisms to get away from it [79]. By identifying and targeting the unique mechanisms of resistance in each patient's tumor, precision medicine can help to adapt therapies more effectively, overcome resistance, and improve treatment outcomes.

### 6.2 The Phenomenon of Heterogeneity

The former is due to intra-tumor heterogeneity while the latter is as a result of clonal evolvement, which contributes to complex issues regarding drug resistance in precision medicine. There may be diverse reactions upon therapeutic intervention because a neoplasm has heterogeneous subpopulations of cells, each having unique genomic profiles. Tumor heterogeneity reflects several levels of complexity that make it important for novel therapeutic approaches to address it integrally [80].

## 7 ETHICAL AND PRIVACY CONSIDERATIONS: PROTECTION OF GENOMIC DATA

### 7.1 The Importance of Informed Consent in the Context of Counseling

The ethical issues related to precision medicine have mainly to do with informed consent and genetic counseling. Genomic profiling patients may come across unexpected incidental findings, such as genetic information that does not relate to the main aim of the test. Hence, it is important to have a prioritized comprehensive pre-test counseling and explicit procedures should be put in place for managing incidental findings, as emphasised by ethical considerations [81].

### 7.2 The Significance of Data Security and Privacy in Contemporary Society

There are significant privacy concerns associated with the storage and sharing of genomic data. Genomic information is intrinsically sensitive, and the possibility of re-identification threatens patient privacy. The use of strong data protection strategies such as encryption and anonymization is the key to preserving trust from people contributing to genomic databases and research initiatives [82].

To fully realize the potential of precision medicine, it is imperative to address economic obstacles to access, improve our ability to interpret intricate genomic information, uncover mechanisms of resistance, and effectively navigate ethical and privacy concerns. Furthermore, to effectively navigate the complex landscape of precision medicine, it is imperative to foster collaborative endeavors among scientific, medical, and ethical disciplines as the field continues to progress [83].

## CONCLUSION

Cancer research and treatment are bound to undergo revolutionary changes as precision medicine develops further. The development of single-cell sequencing has allowed us to understand the complexities of cancer at a level of detail never possible before. It reveals the genetic makeup of individual cells, providing insight into subpopulations exhibiting unique molecular profiles. This fine-grained knowledge is extremely promising for customizing therapies in terms of knowing the unique genetic composition of every single cell, potentially resolving intra-tumor heterogeneity issues. Combining targeted drugs with complementary mechanisms of action has the potential to thwart resistance mechanisms and improve treatment efficacy. This strategy goes beyond conventional chemotherapy regimens and is expected to flourish at the nexus of innovative technology, well-planned therapeutic combinations, and preemptive preventive care. Concurrently, the incorporation of precision care with these developments into prophylactic approaches presents the possibility of detecting cancer in its most amenable stages. Nevertheless, as a research venture into these unexplored areas, a brief caution is required, addressing moral issues and making sure that all, regardless of their economic background, can benefit from precision medicine.

## Figures and Tables

**Fig. (2):**
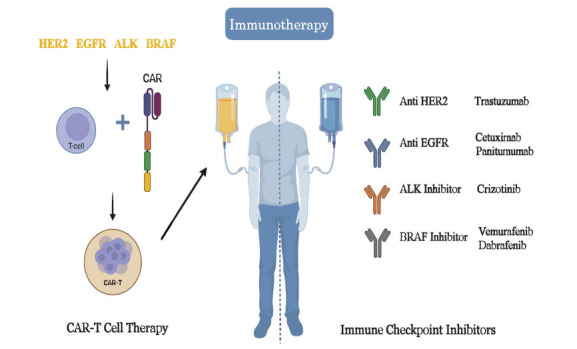
Immunotherapy and precision medicine.

## LIST OF ABBREVIATIONS

ALK: Anaplastic Lymphoma Kinase
CAR-T: Chimeric Antigen Receptor T-Cell
CARs: Chimeric Antigen Receptors
CTCs: Circulating Tumor Cells
ctDNA: Circulating Tumor DNA
CTLA-4: Cytotoxic T-Lymphocyte-Associated Protein 4
EGFR: Epidermal Growth Factor Receptor
HER2: Human Epidermal Growth Factor Receptor 2
ICIs: Immune Checkpoint Inhibitors
mCRC: Metastatic Colorectal Cancer
MEK: Mitogen-Activated Protein Kinase
NGS: Next-Generation Sequencing
NSCLC: Non-Small Cell Lung Cancer
PD-1: Programmed Cell Death Protein-1
PD-L1: Programmed Death-Ligand 1
TMB: Tumor Mutational Burden
VEGF: Vascular Endothelial Growth Factor
WGA: Whole Genome Amplification
WTS: Whole Transcriptome Sequencing
